# Motor Recovery After a Hemispherectomy: Review of Mechanisms and the Potential of Neuromodulation to Enhance Motor Outcomes

**DOI:** 10.1177/08830738251413830

**Published:** 2026-02-13

**Authors:** David Bergeron, Dorothy Barthélemy, Aristides Hadjinicolaou, Marco Bonizzato, Marina Martinez, Numa Dancause, Alexander G Weil

**Affiliations:** 1 Division of neurosurgery, 5622 Université de Montréal, Montreal, Quebec, Canada; 2 459878 Centre for Interdisciplinary Research in Rehabilitation of Greater Montreal (CRIR) and Centre Intégré Universitaire en Santé et Services Sociaux (CIUSSS) du Centre-Sud-de-l'Île-de-Montréal, Montreal, Quebec, Canada; 3 Division of Neurology, Department of Pediatrics, 25461 Sainte-Justine University Hospital Centre, Montreal, Quebec, Canada; 4 Department of Electrical Engineering and Institute of Biomedical Engineering, 5596 Polytechnique Montréal; Mila - Québec AI Institute, Montreal, Quebec, Canada; 5 Département de Neurosciences and Centre interdisciplinaire de recherche sur le cerveau et l'apprentissage (CIRCA), 5622 Université de Montréal, Montreal, Quebec, Canada; 6 Département de neurosciences, Faculté de médecine, 5622 Université de Montréal, Montreal, Quebec, Canada; 7 Division of Neurosurgery, Department of Surgery, Sainte-Justine University Hospital Centre, Montreal, Quebec, Canada

**Keywords:** hemispherectomy, neuromodulation, neuroprosthesis, rehabilitation, spinal cord stimulation

## Abstract

In children with severe, refractory hemispheric epilepsy syndromes, the removal or disconnection of the diseased cortex on one hemisphere from the rest of the brain (hemispherectomy) is a last-resort treatment to cure epilepsy. The removal or disconnection of the motor cortex expectedly leads to contralateral hemiparesis. Partial recovery of the leg or proximal arm may occur over time from the plasticity of alternate motor pathways, but finer hand movements generally do not recover. The advent of neuroprostheses delivering invasive or non-invasive stimulation at different levels of the motor pathways holds promise to enhance motor recovery after a neurologic injury. In this manuscript, we review the mechanisms of motor recovery after a hemispherectomy and discuss how emerging neuromodulation options could be used to improve function. We conclude that the most suitable neuromodulation options for short-term clinical trials are vagal nerve stimulation paired with rehabilitation, and tonic spinal cord stimulation (transcutaneous or with implanted electrodes). We also identify promising neuromodulation options that would require further preclinical investigation in animal models: subcortical deep brain stimulation (motor thalamus, contralateral dentate nucleus), brain-spine interfacing, and motor cortex stimulation. Altogether, this manuscript lays the theoretical foundations for the investigation of neuromodulation therapies to improve the motor outcomes of patients who underwent a hemispherectomy for refractory epilepsy.

In children with refractory hemispheric epilepsy syndromes, the removal or disconnection of the diseased cortex on one hemisphere from the rest of the brain (hemispherectomy) is a last-resort treatment to cure or control epilepsy.^1^ However, the removal or disconnection of the motor cortex expectedly leads to contralateral hemiparesis. Partial recovery over time may occur. Patients who had hemispherectomy in childhood can attain high levels of cognitive and sensorimotor abilities.^2^ Research on different animal models of hemispherectomy, as well as functional imaging in patients who underwent hemispherectomy, has helped to uncover the mechanisms of motor recovery, notably the plasticity in the contralateral, intact cortex and the subcortical motor pathways.^3^ The advent of neuroprostheses delivering invasive or non-invasive stimulation at different levels of the motor pathways holds promise to enhance motor recovery in patients with brain injuries, such as hemispherectomy.^4–7^ In this article, we review the mechanisms of motor recovery after a hemispherectomy and discuss how emerging neuromodulation options could be used to improve function the surgery.

## Motor Outcomes Following Hemispherectomy, and Factors Associated With Recovery

Multiple studies have reported on the motor and functional outcome of patients who underwent hemispherectomy.^1,8–28^ In the largest cohort to this date, Moosa et al^2^ reported on 115 children who underwent hemispherectomy, with a mean follow-up of 6 years. All but 1 patient had some pre-operative motor deficits, and 64% had severe hemiparesis. The hemiparesis remained unchanged in 54%, worsened in 36% and improved in 10%. Eighty-three percent of patients could walk independently at follow-up. Patients with Rasmussen encephalitis were more likely to report worsening of the hemiparesis after surgery (7/10, 70%), as opposed to 33% for other etiologies. Structural abnormalities in the non-operated hemisphere on magnetic resonance imaging (MRI), pre-existing bilateral motor deficits, and post-operative seizure recurrence were associated with inability to walk independently. These results are concordant with other studies, in which hemiplegia worsened post-operatively in 20% to 50% of patients.^12–14,24–27^

In imaging studies, pre-operative atrophy of the cerebral peduncle ipsilateral to the hemisphere removed was associated with a lower risk of worsening hemiparesis.^17,20,21^ This suggests that long-standing pre-existing damage to the corticospinal tract on the side of the hemispherectomy has already led to reorganization of motor pathways. If this corticospinal tract is not already atrophic pre-operatively, it becomes atrophic post-operatively by Wallerian degeneration.^29^ Post-operatively, the size of alternate descending motor pathways like the rubrospinal, reticulospinal tracts (estimated from fractional anisotropy measurements at diffusion MRI) correlates with proximal motor recovery of the paretic limb. Re-organization of these alternate subcortical tracts does not appear sufficient to support dexterous hand movements. Preservation of hand function is rather predicted by the pre-operative size of the ipsilateral pyramidal tract.^30^

Although most patients recover independent walking over time, they display gait alterations such as hip circumduction, briefer paretic limb stance and slower walking speeds, which can improve with rehabilitation.^10^ Pre-operative rehabilitation may also help improve locomotor outcomes.^31,32^ Regarding the arm and hand, most patients do not recover grasping ability or fine hand movements, even with intensive rehabilitation.^33^ In Küpper et al,^33^ 30 of the 50 patients who had some ability to grasp with their paretic hand lost grasping ability after the surgery; conversely, 5 of the 52 patients who could not grasp prior to the surgery developed the ability to grasp post-operatively. Overall, only 25 of 102 patients could grasp after hemispherectomy, after a 1-4 years’ follow-up; all these 25 patients suffered from pre- or perinatal brain lesions.^33^ This suggests that re-organization of an uncrossed corticospinal pathway from the intact hemisphere had already occurred before the hemispherectomy and these pre-procedure changes are important to increase the chances of preserving dextrous hand movements.

Functional MRI studies have shown that many patients prior to hemispherectomy have a larger motor representation of ipsilateral movements in the intact hemisphere. Furthermore, the activity related to the paretic hand increases in the intact hemisphere following the hemispherectomy.^3,10,11,34–38^ The somatotopy of motor representations of the paretic hand is similar to that of movements of the unaffected contralateral hand.^36,39^ The anatomical proximity of representations for the right and left limbs in the intact cortex may explain the occurrence of mirror movements in children who underwent hemispherectomy, wherein voluntary movements of the intact limb cause concomitant involuntary movements in the paretic limb.^40^ Presumably, cortical motor neuron populations aiming at the movement of a single limb inadvertently cause the movement of both limbs, because of abnormal synaptic sprouting and re-organization of bilateral projections that could occurred after the early damage to the injured hemisphere.^41^

In patients who underwent hemispherectomy, the return of voluntary movements can only occur through motor commands originating in the remaining hemisphere. Therefore, we will review the tracts and mechanisms by which motor commands from this intact hemisphere can reach the ipsilateral, paretic limb. These tracts are good candidates to target for neuromodulation that enhance motor outcomes.

## Important Tracts and Pathways for Ipsilateral Cortical Motor Control in Animals and Humans

### Ipsilateral Corticospinal Tract

Initially thought to specifically represent the direct corticomotoneuronal connection from the primary motor cortex (Betz cell from layer V of primary motor cortex [M1]) to motoneurons of the anterior horn, the corticospinal tract is now defined more broadly as the efferent motor connections from the cortex to the spinal cord. These motor projections can originate from other areas than M1, for example, from premotor frontal areas, cingulate areas and parietal cortex, and form dissynaptic connections with the anterior horn through spinal interneurons.^42^ The development of direct corticomotoneuronal connections from M1 to motoneurons of the anterior horn correlates with forelimb and hand dexterity across species.^42–44^ Furthermore, the post-natal development and myelination of this direct pathway coincide with the capacity to perform dexterous movements in primates.^45–49^ At birth, corticospinal neurons form immature collaterals at almost every spinal level, without forming direct connections in the ventral horn.^45,48,49^ In the first 8 months of postnatal development, direct corticomotoneuronal synapses are formed in the contralateral ventral horn, while immature collaterals are eliminated.^45,48–51^ At the end of this pruning process, the corticospinal tract becomes a primarily crossed pathway, with 80% to 95% of the axons crossing to the contralateral side at the cervicomedullary junction,^52–54^ and about 10% of the corticospinal projections reaching ipsilateral targets in the spinal cord, via the ventromedial and dorsolateral ipsilateral corticospinal tracts.^53,54^ In addition, some corticospinal axons were shown to cross the spinal cord midline (second decussation) to terminate in the ipsilateral medial motor pool in different animal models.^52,54,55^ In individuals with unilateral corticospinal damage sustained early in development, activity-dependent plasticity prevents the pruning of immature ipsilateral synapses, resulting in stronger ipsilateral corticospinal transmission.^51,52,56–62^ When the damage is sustained after the critical pruning period the potential for increased ipsilateral corticospinal transmission is more limited.^58,59,63^ As a result, children with congenital hemispheric damage have strong ipsilateral responses to transcranial magnetic stimulation (TMS) applied to M1, presumably from unpruned ipsilateral corticospinal projections.^59^ Staudt et al^59^ noted that children with prenatal hemispheric damage recover useful contralateral hand function when the damage is sustained in the first or second trimester of pregnancy. These children with good hand function have high-amplitude and low-latency ipsilateral responses to TMS.^39^ In comparison, children with lesions acquired later in pregnancy (late-third trimester) or postnatally show higher-latency and lower-amplitude ipsilateral responses to TMS,^39^ and generally do not recover grasping ability or fine hand movements.^59^ Neurotypical children have ipsilateral responses to TMS in upper extremities up to 10 years of age, with higher latency and lower amplitude than children who sustained early hemispheric damage.^64^ After age 10 years, ipsilateral responses considerably decrease,^64^ even in patients who had a stroke.^65^ Nevertheless, at higher amplitudes, TMS may elicit some high-latency, low-amplitude ipsilateral responses in adults.^66–68^

### Rubrospinal Tract

The rubrospinal tract originates from the magnocellular division of the red nucleus in the mesencephalon, decussates within the ventral midbrain tegmentum, and projects to spinal cord interneurons, modulating the flexor activity of the contralateral arm and leg.^69,70^ Conversely, the parvocellular division of the red nucleus relays information from the motor cortex to contralateral dentate nucleus of the cerebellum through the inferior olivary complex, known as the triangle of Mollaret.^71^ The red nucleus and contralateral dentate nucleus are also densely connected to the motor thalamus (ventral anterior, ventral intermediate).^69^ The magnocellular red nucleus receives ipsilateral projection from cortical neurons in prefrontal and parietal areas, which forms the cortico-rubrospinal system.^72,73^ Early in development, the cortico-rubral pathway is bilateral, with crossed cortico-rubral pathway being pruned during post-natal development; when cortical damage is sustained early in development, the crossed cortico-rubral pathway evades pruning and remains as an alternate pathway for ipsilateral motor control of the preserved cortex towards the affected limbs.^74^ In Lawrence and Kuypers’s studies, the subsequent lesioning of lateral brainstems pathways, mainly the rubrospinal tract, in animals who recovered from bilateral lesioning of the corticospinal tract reinstated grasping and climbing deficits, without affecting walking.^75,76^ Although its role is considered limited in humans, in relation to the development of the corticospinal tract,^77,78^ the cortico-rubrospinal tract has the potential to compensate for motor function in cases of injury of the corticospinal tract. Notably, patients who have a stroke in cortical motor areas exhibit increased compensatory activity in the bilateral red nuclei, as well as increased fractional anisotropy of the rubrospinal tracts, which correlates with the degree of mobility recovery.^79–82^

### Cortico-reticulospinal Pathway

The reticulospinal tract originates from the pontine and bulbar reticular formation and reaches spinal interneurons regulating gait, postural control, muscle tonus, and locomotion.^83–85^ It has a medial component originating from the pontine reticular formation (ventromedial funiculus), controlling movements of distal extremities; and a lateral component (ventrolateral funiculi), originating from the bulbar reticular nucleus, regulating proximal muscle movements. The reticular formation is a midline structure receiving inputs from both hemispheres, with collaterals originating mostly from the premotor cortex.^86,87^ It projects to the spinal cord bilaterally,^83,88,89^ mainly to spinal interneurons. However, reticulospinal neurons with direct motoneuronal synapses were reported in animal models.^90^ As the reticular formation receives extensive converging inputs from both hemispheres, the redundancy of function is a potential mechanism of recovery after hemispheric damage.^87^ Because of its pattern of innervation, the reticulospinal tract is thought to have a primary role in postural stability and stereotyped, multi-segmental movements such as walking.^75,84,91^ In 1968, Lawrence and Kuypers investigated the role of extra-pyramidal systems in motor recovery after corticospinal injury: after bilateral lesioning of the corticospinal tract, animals initially suffered flaccid paralysis, followed by gradual recovery of walking and climbing ability, but never recovered fine hand movements.^75,76^ In the recovered animals, cutting the medial brainstem descending systems (reticulospinal and vestibulospinal) produced severe impairment of gross movements, without impairing grasping.^75^ In addition, Zaaimi et al^92^ used micro-stimulation and intercellular recordings to assess the contribution of the uncrossed lateral corticospinal tract and reticulospinal tract to recovery of affected hand movement after stroke in non-human primates. Stimulation of the medial longitudinal fasciculus elicited bilateral responses, which were enhanced after lesioning the contralateral corticospinal tract. In contrast, stimulation of the uncrossed lateral corticospinal tract rarely elicited responses before or after lesioning. These findings reinforce the idea that ipsilaterally descending subcortical pathways may have a more prominent contribution to paretic limb movement than the uncrossed lateral corticospinal tract. In chronic stroke patients, fractional anisotropy within all extrapyramidal tracts located throughout the brainstem, especially the reticulospinal tract, correlates to motor function of the lower limb. This is in line with the notion of increased reliance on extrapyramidal pathways to support motor function after corticospinal tract damage.^93–96^

### Vestibulospinal Tract

Originating from the lateral vestibular (Deiter's) nucleus, the lateral vestibulospinal tract travels ipsilaterally to reach spinal interneurons, regulating extensor tone and postural adjustments of limbs to movements; medial vestibulospinal tract originates from the medial vestibular nucleus and descends bilaterally to reach cervical interneurons, adjusting head position to movements.^97,98^ Although its role in ipsilateral motor control after hemispheric injury is less studied, the lateral vestibulospinal tract is thought to contribute to spinal interneuron hyperreactivity in spasticity after stroke.^99^

### Spinal Interneurons

The direct connections from descending tracts to lower motoneurons in the anterior horn are exclusively found within the corticospinal system.^100^ All other extrapyramidal pathways involved in motor recovery after corticospinal damage first synapse onto interneurons, which themselves activate anterior horn motoneurons. More specifically, a population of interneurons known as the propriospinal system integrate and modulate ascending and descending signals.^101,102^ Interneurons subdivide into a minimum of 11 classes based on their developmental origin, gene expression, and anatomical projection pattern. These classes comprise 6 dorsal classes (dI1-6) and 5 ventral classes (V0, V1, V2a, V2b, and V3).^103,104^ V0 and V3 are commissural interneurons, which allow interlimb coordination during locomotion and other movements. V1 (Renshaw) are inhibitory interneurons that establish a negative feedback system regulating the firing rate of the motor. V2a (glutamate) and V2b (GABA) interneurons are essential to both forelimb and hindlimb movements. More specifically, V2a interneurons represent a population of excitatory interneurons within the ventral spinal cord, connecting extrapyramidal descending tracts (such as reticulospinal) with anterior horn motoneurons. They were revealed to be the main neuronal population driving locomotor recovery with spinal cord stimulation following spinal cord injury.^105^ As such, they represent an important neuromodulation target to amplify residual descending tracts’ modulation of movement.^106,107^

Given these pathways for ipsilateral voluntary motor control, we will then review the mechanisms of recovery, which may be enhanced by neuromodulation.

## Mechanisms of Recovery

### Preservation of Uncrossed Corticospinal Projections From the Intact Hemisphere to the Paretic Limbs That Evaded Pruning

In animal models of hemispherectomy, the “Early Lesion Effect” (Kennard principle)^108^ is still widely accepted. Following this principle, recovery from hemispheric damage is greater when the damage occurs before the pruning of superfluous connexions, either prenatally or in the early post-natal period. For instance, in rats, optimal recovery after hemidecortication is obtained when the surgery is performed at about 7 days after birth (P7), or a few days after.^109^ These rats display aberrant ipsilateral (uncrossed) corticospinal projection originating from the undamaged hemisphere.^56,57,60,62,110–112^ Sprouting of collaterals along undamaged corticospinal projections to the thalamus, the striatum, the red nuclei, and the pontine nuclei bilaterally is observed, indicating bilateral control of cortico-rubrospinal and cortico-reticulospinal pathways by the undamaged hemisphere.^113–117^ Moreover, there is evidence of re-crossing of corticospinal neurons within the spinal cord near their level of termination (double-crossing).^55,118^ As a result, stimulation of the undamaged motor cortex of neonatally hemidecorticated rats induces low-threshold ipsilateral forelimb movements, which is not observed in animals who underwent hemidecortication after more than 30 days post-natally.^112^ In rats, uncrossed (ipsilateral) corticospinal tract fibers develop within the first week of life and reach the lumbar level at around week 7, then sequentially disappear from the upper lumbar, lower thoracic, and middle thoracic spinal levels through synaptic pruning.^119^ Bilateral motor control in neonatally hemidecorticated rats is hence thought to be mediated by uncrossed (ipsilateral) corticospinal neurons that escaped synaptic pruning or neuronal apoptosis^56,110^ and by sprouting of collaterals from the decussated corticospinal tract (spinal re-crossing, bilateral subcortical projections) which is facilitated when the myelination of corticospinal axons is not yet completed.^120^ These mechanisms of unihemispheric bilateral motor control with neonatal damage to one hemisphere were also described in cats^121–125^ and non-human primates.^52,54,126^ In rhesus monkeys, the best outcome of neocortical damage occurs when the resection is performed by the end of the second trimester of gestation.^127^ Regarding synaptic pruning, there is rapid pruning between 2 months and 2.5 years postnatally in non-human primates, with further loss stopping by about 4 years of age.^128,129^ Since the crucial maturational period for corticospinal pruning and myelination concludes quite early in children, these mechanisms are improbable contributors to recovery following hemispherectomy. However, they likely play a role in the recovery from perinatal and early post-natal damage. This could clarify why patients with perinatal injuries, exhibiting significant pre-operative asymmetry in cerebral peduncles, experience less motor deterioration after hemispherectomy, as they rely on ipsilateral corticospinal pathways that escaped apoptosis long before the hemispherectomy.^21^ This can be measured with TMS applied to the intact hemisphere. In children with congenital hemispheric damage, strong ipsilateral responses to TMS in the primary motor cortex (M1) have been observed, whereas such responses are not observed in neurotypical children of the same age. These responses have a short and a longer latency responses, presumably originating from direct ipsilateral corticospinal transmission and cortico-reticulospinal transmission, respectively.^34,59,64,130–132^

### Increased Reliance on Extrapyramidal Motor Tracts and Spinal Cord Plasticity

In patients with later acquired brain damage prior to hemispherectomy, ipsilateral responses evoked by TMS applied to M1 have longer latencies, presumably through polysynaptic cortico-reticulospinal pathway.^39^ The cortico-reticulospinal pathway has the potential for reorganization.^133^ This pathway has been suggested to be the main driver of walking recovery with readaptation following a spinal cord injury.^105,133–135^ It has also been suggested that the reticulospinal pathway has a greater potential for plasticity and re-organization than the corticospinal pathway.^135^ In rats, it was shown that animals with cervical spinal cord hemisection have reticulospinal fibers from the intact hemicord at cervical and lumbar levels, a phenomenon not observed for corticospinal or vestibulospinal tracts.^135^ Intraspinal circuits, including propriospinal fibers, can relay supraspinal inputs to enable recovery after stroke or spinal cord injury.^101,136^ These circuits also exhibit plasticity, characterized by mechanisms such as synaptogenesis and axonal sprouting.^137^ This reorganization occurs both in the spinal cord, involving both spinal re-crossing and propriospinal connections, and in the brain, including cortico-striatal and cortico-brainstem connections.^118,138,139^

### Cortical-Subcortical Reorganization and Synaptic Plasticity

Two primary types of synaptic plasticity have been described: Hebbian plasticity and homeostatic plasticity. Hebbian plasticity is a positive feedback-mediated form of plasticity in which synapses between presynaptic and postsynaptic neurons that are coincidently active are strengthened. Homoeostatic plasticity is a negative feedback-mediated form of plasticity maintaining network activity in case of reduced stimulation.^140^ Intensive rehabilitation presumably reinforces synaptic strength in residual motor pathways through Hebbian plasticity mechanisms.^140–142^ Conversely, certain neurons that have lost their connections due to hemispheric damage are at risk of experiencing a decline in synaptic strength, potentially leading to apoptosis. Homeostatic plasticity acts as a protective mechanism by sustaining a basal level of synaptic activity despite reduced input, thereby safeguarding these neurons from degeneration.^140^ These synaptic plasticity mechanisms affect the strength of synaptic connections that have reached their target. Other proposed mechanisms include the sprouting of axons towards new target regions or sprouting of axonal terminals in axons traversing nearby deafferented regions.^143–145^

In animals with large stroke or hemispherectomy, motor cortical maps undergo changes in the ensuing weeks, correlating with the recovery of motor functions.^146,147^ Most likely, this occurs because of sprouting of synaptic terminals and of axons over short distances, in residual neurons already performing similar functions.^148,149^ In terms of motor function, it is increasingly recognized that the primary motor cortex, in addition to its classically described somatotopic organization, often referred to as the Penfield motor homunculus, in fact contains representations of all 4 limbs,^150^ notably through a somato-cognitive action network.^151,152^ In neurotypical adults, the somato-cognitive action networks within the motor cortex include bilateral motor representations for proximal limbs, contrasting with mostly unilateral representation of fine distal extremities like fingers movements.^151^ Likewise, about 20% of the corticospinal neurons in the supplementary motor area neurons project to the ipsilateral cord.^153^ The increased activity and synaptic boutons of these residual ipsilaterally projecting neurons already achieving the impaired function likely explain most of the recovery of the paretic hand.

Nevertheless, there are some reports of long-distance re-wiring of neurons after neurologic injury. For instance, in adult rats that sustained unihemispheric damage, Napieralski et al^139^ reported the intact cerebral cortex sprouting crossed axonal projections to the striatum of the injured side of the brain. It is unclear whether this kind of long-distance cortical-subcortical re-wiring occurs in humans past the critical neurodevelopmental period. We know that the ipsilesional subcortical nuclei (basal ganglia, thalamus) undergo rapid atrophy from deafferentations after hemispheric damage in humans and animal models.^22,154^

In terms of potentiating this cortical plasticity and re-organization, repeated cortical microstimulation can accelerate the re-organization of cortical representation of movements in adult rats.^155^ Likewise, pharmacologic intervention, or stimulation of neural structures connected to a widespread monoaminergic network (such as the vagal nerve), can potentiate the molecular mechanisms involved in neuronal and synaptic plasticity.^156–159^

## Neuromodulation Targets to Improve Motor Recovery After Hemispherectomy

**Figure 1.**
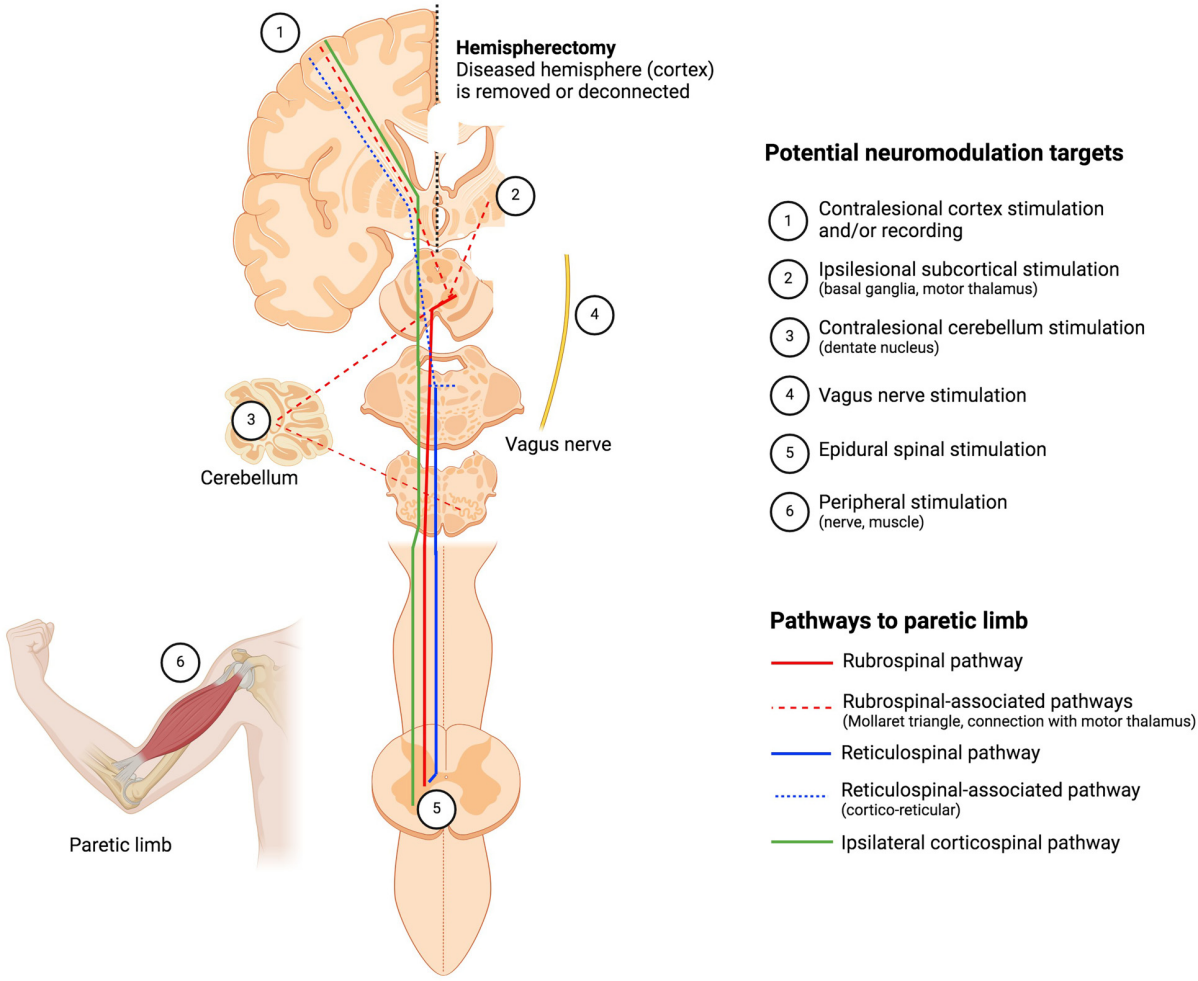
Summary of pathways to the paretic limb and potential neuromodulation targets in patients who underwent a hemispherectomy for refractory epilepsy. The figure shows a coronal representation of a brain with the cortex removed on one hemisphere (in patients, either removed or disconnected), yet with preserved subcortical structures (thalamus, basal ganglia, etc). Neuronal pathways to the paretic limb are represented, namely, the ipsilateral corticospinal pathway (green), the rubrospinal pathway (red), and the reticulospinal pathway (blue). Associated pathways are shown as dashed lines, such as the cortico-rubral pathway, the cortico-reticular pathway and the Mollaret triangle. The Mollaret triangle represents the connection of the red nucleus with the inferior olive (through the central tegmental tract), itself connected to the contralateral dentate nucleus (through the inferior cerebellar peduncle), back to the red nucleus through the decussating superior cerebellar peduncle; this network is connected with the motor thalamus (ventral anterior, ventral intermediate). The potential neuromodulation targets are also represented. The figure was produced using the Biorender app (https://app.biorender.com/) (Color figure online).

Drawing upon our understanding of the pathways and mechanisms involved in motor recovery after a hemispherectomy, we now review various neuromodulation techniques and neuroprosthetic approaches aimed at enhancing immediate and long-term motor function in children undergoing this procedure (summarized in Figure 1).

### Spinal Cord Stimulation

Spinal cord stimulation modulates spinal circuits to produce specific muscular synergies depending on the electrode placement and simulation parameters.^160,161^ Particularly, electrical epidural stimulation administered to the lumbosacral cord has emerged as a potent technique for modulating leg movements and potentially aiding in the resurgence of descending motor commands.^162,163^

### Tonic Spinal Cord Stimulation

Tonic spinal cord stimulation modulates the excitability of spinal locomotor circuits^164^ and may facilitate the recruitment residual supraspinal inputs to spinal motoneurons.^165^ Tonic spinal cord stimulation primarily targets the primary sensory afferents in the dorsal columns and dorsal roots, which are known to convey excitatory inputs to motoneurons and interneurons throughout the spinal cord.^161,165,166^ Most animal and human studies have employed epidural spinal stimulation as an adjunctive approach to enhance the effects of motor training. Studies employing tonic stimulation in individuals with incomplete spinal cord injury found that the application of tonic stimulation following intensive physical training may aid in regaining independent volitional control of movements and treadmill walking with body weight support, but only when the stimulation is turned on (orthotic effect).^162,167^ Stimulation integrated into tasks involving volitional motor commands, such as overground walking, is pivotal for recovery after spinal cord injury.^162,163,168–170^ In patients with hemiparesis caused by stroke, tonic cervical stimulation improves the strength and precision of hand movements during rehabilitation, increasing the excitability of interneurons to a reduced supra-spinal input.^4,166,171^ Spinal cord stimulation can also be performed with non-invasive transcutaneous delivery.^172,173^ However, transcutaneous spinal cord stimulation lacks the spatial specificity of stimulation and may be impractical for chronic use outside of rehabilitation settings. Nevertheless, when combined with intensive rehabilitation, it could assist in promoting motor recovery in patients with spinal cord injury.^172,174,175^ Early clinical studies have shown rehabilitation benefits of transcutaneous spinal cord stimulation in patients with spinal cord injury (SCI)^175^ and children with cerebral palsy.^176^ No data are currently available regarding transcutaneous spinal cord stimulation in patients with stroke.

### Phasic Spinal Cord Stimulation—Brain-Spine Interface

Phasic stimulation of posterior large-diameter afferents of the spinal cord generates reflex movements in the corresponding myotome. The sequential stimulation of different nerve roots, in a precise spatial and temporal manner, can generate functional movement sequences such as walking. Spatiotemporal phasic spinal cord stimulation has shown superiority over continuous, unpatterned stimulation in reinstating locomotor movements and specific muscular synergies.^177,178^ In individuals with paraplegia from spinal cord injuries, pulses of epidural stimulation applied to the posterior lumbosacral roots can elicit anti-gravity movements of the legs. A sequence of spatiotemporal stimulations could facilitate weight-bearing and leg movement sequences necessary for locomotion.^5,178^ Patients could additionally adjust the magnitude of movements through their own volition, and some could eventually regain the ability to generate movements without stimulation.^5^ The stimulation sequence could be triggered either manually by the push of a button^5^ or automatically through the decoding of brain activity (brain-spine interface).^7^ Decoding movement intention with epidural or subdural electrodes is relatively straightforward in patients who have an intact brain, with an intact representation of their arms and legs within the primary motor cortex.^7^ However, it becomes more challenging in patients whose primary motor representation has been removed or disconnected. The motor representation of the paretic limb in the intact hemisphere (ipsilateral corticospinal tract) increases after a hemispherectomy.^10^ However, real-time decoding of ipsilateral movement intentions has never been attempted for clinical applications. In patients with relatively preserved pre-operative motor function, who undergo a functional hemispherectomy, it may be possible to decode motor intentions directly in the disconnected cortex. However, the interruption of cortico-thalamic loop and the ascending reticular input to the cortex leads to a marked reduction in spontaneous cortical activity, measured with EEG^179^ and cerebral blood flow at arterial spin labelling MRI, resembling a state of impaired consciousness.^180^ Even if motor intentions may be decoded in the disconnected cortex, it is unclear whether this activity would be stable over time, as the disconnections of these neurons from their effector targets and most of their afferents would likely lead to a reduction of their activity or even their apoptosis. Regarding arm movements, it has been shown that single pulse epidural stimulations of dorsal root afferents in the cervical spinal cord can elicit anti-gravity arm movements.^171,181^ However, because of the overlapping innervation of forearm and hand muscles, the selective facilitation of individual finger muscles is challenging with epidural implants alone.^171,181^

### Functional Electrical Stimulation—Brain-Muscle Interface

As cervical epidural spinal stimulation may not be optimal to restore fine hand movements, stimulation can also be applied closer to the effector, that is, in peripheral nerves using intraneural electrodes^182,183^ or directly in the muscle with functional electrical stimulation (FES). These stimulations have been linked with decoding of motor intentions in the cortex (brain-muscle interface) to restore some functional hand movements in patients with tetraplegia^184–186^ and stroke.^187^ Overall, even if we achieve an accurate decoding of movement intentions of the arm and hand, it remains unclear whether an optimal combination of phasic stimulations (epidural spinal, peripheral nerve, FES of muscle groups) could produce satisfactory, functional movements of the upper extremity for the long-term restoration of functional movements in daily life.

### Vagus Nerve Stimulation

Vagus nerve stimulation (VNS) is a promising therapeutic approach currently employed in clinical practice to address a range of neurologic conditions, such as epilepsy,^188^ depression^189^ and stroke,^6^ presumably through retrograde activation of brainstem nuclei, causing a timed burst of neuromodulators impacting brain-wide excitability levels and enhancing neural plasticity.^190–193^ Notably, this method has recently gained approval from the Food and Drug Administration as the first neuromodulation therapy to treat motor deficits resulting from chronic stroke.^6^ In a pivotal controlled study involving 106 patients, a clinically meaningful response on the Fugl-Meyer Assessment-Upper Extremity score was achieved in 23 of 53 patients in the VNS group compared with 13 of 55 patients in the control group.^6^ This effect was corroborated by several smaller studies.^194^ Additionally, VNS can be administrated non-invasively during rehabilitation, although its efficacy in this context may be reduced.^194,195^ Contrary to VNS for epilepsy, where chronic intermittent stimulation is delivered, VNS for rehabilitation has to be applied precisely during movement execution during rehabilitation.^194^ Recent studies suggest that VNS should not only be administered during ongoing movements but specifically following successful movements. In a rodent study, VNS enhanced skilled reach learning, but this enhancement occurred only when VNS was applied after a successful reach completion, revealing that VNS is most effective when linked to rewards, rather than when delivered for every movement attempt or when applied randomly.^196^ Paired, behaviourally coherent VNS, unlike open-loop VNS, is also effective in facilitating the enlargement of cortical motor maps.^192,197^ Some patients who undergo a hemispherectomy have a VNS for seizure control, but VNS has never been attempted to improve motor recovery in the post-operative period.

### Subcortical Deep Brain Stimulation

Similar to VNS, deep brain stimulation (DBS) of subcortical regions has the potential to modify neuronal excitability and plasticity during rehabilitation.

#### Cerebellar DBS

In a recent study, DBS of the contralesional cerebellar dentate nucleus during rehabilitation promoted functional reorganization of ipsilesional cortex in 12 individuals with moderate to severe upper-extremity impairments from stroke.^198^ In a rat model of stroke, dentate nucleus stimulation combined with motor training enhanced cortical plasticity compared with motor training alone, with greater perilesional reorganization, and increased perilesional expression of synaptic markers of long-term potentiation and plasticity.^199–201^ Cerebellar stimulation was never attempted in animal models of hemidecortication or large cortical stroke. As there is no ipsilesional residual cortex to modulate, it is unclear if contralesional cerebellar stimulation would have any effect at all, perhaps on residual pathways to the affected limb (reticulospinal, rubrospinal, and uncrossed corticospinal).

#### Thalamic DBS

Stimulation of the motor thalamus facilitates the recruitment of cortico-spinal neurons within the motor cortex, which in turn increases motor output in intact upper extremities and paretic limbs after lesions of the corticospinal tract.^202^ In non-human primates, stimulation of the ventral lateral thalamus enhanced the response to internal capsule stimulation, with increased upper-limb motor-evoked potentials (MEPs) and grip forces^202^; notably, stimulation frequencies between 50 and 80 Hz consistently potentiated the MEP amplitudes with sustained outputs. In patients undergoing thalamic DBS implantation for tremor (ventralis intermediate nucleus [ViM]), 50-80-Hz stimulation of the ViM significantly increased MEPs. Finally, 55-Hz ventral lateral thalamus stimulation in a patient with corticospinal damage from stroke improved motor control, notably the control of grip strength.^202^ These results are preliminary, and the effect of thalamic stimulation after hemispherectomy has never been studied. After a hemispherectomy, the ipsilesional thalamus undergoes atrophy from deafferentation, and there is no ipsilesional cortex to modulate; hence, it is unclear whether ipsilesional stimulation would have any effect. Stimulation of the contralesional (or bilateral) motor thalamus may have more effect but may have unwanted effect on the unaffected limb.

### Cortical Stimulation of the Intact Hemisphere

Most of the literature on cortical stimulation for rehabilitation comes from the stroke literature, with the goal of modulating the residual ipsilesional cortex, which does not apply to patients with hemispherectomy. For instance, stimulation applied to the residual ipsilesional cortex resulted in meaningful motor improvements in several animal models of stroke.^203–206^ In humans, epidural motor cortex stimulation of the ipsilesional cortex initially yielded promising results (potentially increased long-term motor recovery)^207,208^; however, the outcomes of a phase 3 trial using epidural motor cortex stimulation in patients with arm paresis from stroke (EVEREST trial) has been negative.^209^ As a non-invasive treatment option, TMS was shown to modulate motor cortex excitability in patients with stroke.^210–212^ Most TMS paradigms have been designed for patients with incomplete hemispheric stroke, with the goal of stimulating ipsilesional perilesional cortex, or inhibiting the contralesional cortex.^157^ In patients with hemispherectomy, there is no perilesional ipsilateral cortex to stimulate, and it would be counter-intuitive to inhibit the only remaining hemisphere, as there is no remaining ipsilesional cortex to disinhibit. Finally, while the risk of seizure is minimal in patients without epilepsy, it is essential to recognize that epilepsy is regarded as a relative contraindication for TMS and cortical stimulation in general.^210^ Stimulation of the contralesional cortex (with the goal of modulating the uncrossed corticospinal tract) may be beneficial but is not supported by an extensive literature. In a rat model of unilateral corticospinal interruption (medullary pyramid), stimulation of the contralesional (intact) corticospinal tract strengthened the connections of the ipsilateral corticospinal tract within the anterior horn of the spinal cord^158^ and improved skilled motor functions of the paretic forepaw.^159^ It is unclear whether the ipsilateral corticospinal tract could be stimulated within the intact hemisphere without causing off-target stimulation to the motor representation of the contralateral (unaffected) limb.

## Roadmap to a Trial of Neuromodulation-Enhanced Rehabilitation After Hemispherectomy

Considering the diverse range of neuromodulation options available for potentially improving motor outcomes in patients with hemispherectomy, we have categorized potential therapies into 2 groups: (1) best suited for exploratory clinical trial; (2) those for which more preclinical research on animal models of hemispherectomy is needed before considering exploratory clinical trials.

### Best Suited for Exploratory Clinical Trial

- **VNS** has recently gained approval from the Food and Drug Administration as the first neuromodulation therapy to treat motor deficits resulting from chronic stroke.^6^ Customizing stimulation parameters to synchronize with rehabilitation (VNS-rehab) could feasibly yield initial findings regarding its impact on ipsilateral corticospinal excitability and long-term motor recovery. Interestingly, some patients who underwent a hemispherectomy already have a VNS implanted to help control their seizures, which would allow for a very low-risk preliminary study. In the landmark VNS-Rehab study, the Fugl-Meyer Assessment Upper Extremity score improved 6 points (pre-specified definition of response) in 47% of patients treated with VNS and 27% of controls.^6^ This relatively modest effect was statistically significant in a cohort of 100 patients with stroke, but given the small number of hemispherectomies performed annually even in highly specialized centers,^213^ reaching statistical power to demonstrate the effect in this population will be difficult.
- **Tonic spinal cord stimulation** has shown efficacy in increasing the excitability of the cervical spinal cord to residual supraspinal inputs in patients with stroke^4^ and could likely help improve the strength of movements relying on ipsilateral corticospinal transmission in patients with hemispherectomy. Using transcutaneous spinal cord stimulation would be a wiser initial approach to gather preliminary data in patients with hemispherectomy. Before advancing to early clinical trials involving implanted epidural electrodes for chronic stimulation in this patient population, it would be advisable to accumulate data on transcutaneous spinal cord stimulation and also gather insights from animal models. This cautious approach is warranted because of the increased likelihood of surgical complications in children who underwent a hemispherectomy. Although currently no device is specifically designed to deliver spinal cord stimulation at parameters targeted towards movement restoration, many groups have used commercially available spinal cord stimulation devices designed for chronic pain, with satisfactory results.^4,167,214,215^

### More preclinical research needed

- **Phasic spinal cord stimulation**, nerve stimulation, and brain-spine interfacing may help restoring movements in patients who underwent a hemispherectomy. However, there remains uncertainty regarding the feasibility of accurately decoding ipsilateral movement intentions in the intact cortex, with the proximity of ipsilateral and contralateral representations (even causing mirror movements with spontaneous cortical activity).^10,36,37,132^ Moreover, it is unclear whether phasic epidural spinal stimulation or peripheral nerve stimulation will yield movements that are sufficiently refined and controlled to provide a clinical benefit. Furthermore, despite early proof-of-concept studies in humans,^7^ there is currently no approved integrated device to allow brain-spine interfacing.
- **Subcortical DBS**, particularly targeting the cerebellar dentate nucleus or the motor thalamus, may help increasing the output and plasticity of the ipsilateral corticospinal pathway and the other alternate pathways towards the paretic limb.^198,202^ However, most studies performed to this date evaluated the effect of stimulation on the residual ipsilesional cortex in patients with partial hemispheric damage. It is unclear whether the ipsilesional thalamus and basal ganglia have any role to play in the recovery of movements after a hemispherectomy: it usually undergoes atrophy from deafferentation but may still receive some inputs from the intact cortex, the ipsilateral red nucleus and contralateral dentate nucleus. It is unclear whether stimulation of the ipsilesional thalamus (or contralateral cerebellum) would have any effect given the changes to thalamic and cerebellar circuitry following hemispherectomy.^216–218^ A deeper basic science understanding of these dynamics is required before considering early clinical trials.
- Motor cortex stimulation, although a theoretically useful strategy, raises important safety concerns, notably (1) the higher risk of causing seizures in the intact hemisphere, as the intact hemisphere may remain more vulnerable to electrical stimulation due to kindling from long-standing epilepsy^219^; and (2) any complication damaging the only remaining hemisphere with invasive implants would have catastrophic consequences. Moreover, most of the preclinical work on motor cortex stimulation focuses on activating the remaining ipsilesional cortex or inhibiting the contralesional cortex, which is not applicable to patients with hemispherectomy. It is unlikely that the ipsilateral corticospinal tract can be stimulated within the intact hemisphere without causing off-target stimulation to the motor representation of the contralateral (unaffected) limb, especially with non-invasive stimulation. Invasive stimulation techniques, including epidural, subdural, and intra-cortical stimulation, have shown promise in achieving selective stimulation of ipsilateral representations.^220^ Notably, intra-cortical stimulation has been successfully tested in animal models of spinal cord injury, demonstrating its potential for clinical translation.^221,222^ However, before considering their application in patients, extensive preclinical research on animal models of hemispherectomy is imperative to establish their safety and efficacy. Caution is warranted because of the elevated risk associated with these interventions.

## Conclusion

We conducted a comprehensive review focusing on the pertinent anatomy and mechanisms underlying motor recovery following a hemispherectomy, along with the potential of innovative neuromodulation therapies to optimize this recovery process. This endeavor will provide a valuable roadmap for initiating exploratory clinical trials and conducting additional preclinical investigations into therapies aimed at enhancing motor recovery in children undergoing hemispherectomy as a treatment for refractory epilepsy.
